# Utility of IMP3, p53, and S100P immunohistochemical stains in distinguishing reactive atypia from dysplasia in cholecystectomy specimens

**DOI:** 10.1186/s13000-024-01550-w

**Published:** 2024-09-27

**Authors:** Evan Sica, Karen T. Shore, Limin Yang, Kara Chan Phelps, Suntrea T. G. Hammer, Purva Gopal, Dipti M. Karamchandani, James Michael Mitchell

**Affiliations:** 1 Department of Pathology, University of Texas Southwestern Medical Center, 5323 Harry Hines Blvd, Dallas, TX 75390-9072 USA; 2 Incyte Diagnostics, PO Box 3405, Spokane, WA 99220-3405 USA

**Keywords:** IMP3, S100P, p53, Gallbladder, Dysplasia, Reactive atypia, Cholecystectomy

## Abstract

**Background:**

Distinguishing reactive atypia from dysplasia in cholecystectomy specimens can be histologically challenging. The aim of this study was to evaluate the utility of IMP3, p53, and S100P immunostains in differentiating reactive atypia from dysplasia in cholecystectomies.

**Methods:**

Fifty-four cholecystectomies were reviewed and characterized into 5 groups: 2 normal, 29 reactive atypia, 16 low-grade dysplasia, 2 high-grade dysplasia, and 5 adenocarcinoma. IMP3, p53, and S100P immunostains were performed and evaluated. IMP3 (nuclear) and S100P (nuclear or nuclear/cytoplasmic) were categorized into negative or positive expression, and p53 was categorized into wild-type and aberrant/mutant expression. Chi-square test was used for statistical analysis.

**Results:**

The patients were mostly middle-aged women (mean 44, range 19–87 years, 81% female), with predominantly Hispanic White ethnicity (80%). The majority of the normal and reactive atypia cases showed negative IMP3 (100% and 75.9%, respectively) and wild-type p53 (100% and 89.7%, respectively) staining. Over half (56.3%) of the low-grade dysplasia and all the high-grade dysplasia cases showed IMP3 positivity. Aberrant p53 staining pattern was seen in half of both low and high-grade dysplasia cases. Adenocarcinoma showed IMP3 positivity in 80% and p53 aberrancy in all cases. S100P showed no statistical significance among the diagnostic categories. Significant differences in staining patterns were found between reactive atypia vs. low-grade dysplasia, and reactive atypia vs. low-grade + high-grade dysplasia using a combination of IMP3 and p53 stains (all *p* < 0.05).

**Conclusions:**

In challenging cholecystectomies, IMP3 positivity or aberrant p53 expression may serve as a useful adjunct to support a diagnosis of dysplasia over reactive atypia.

## Background

Cholecystectomy is one of the most common surgical procedures in the United States, with more than 1.2 million procedures performed annually [1]. As such, gallbladder specimens are routinely sent for histopathological examination. Gallbladder cancer (GBC) is a rare disease with an incidence of 0.5–0.9 per 100,000 people in the US and constitutes almost half of all biliary tract cancers [2]. It carries a poor prognosis with a 5-year survival rate of 5–20%, given its propensity for delayed diagnosis at an advanced or metastatic stage [3, 4]. As the main risk factor for GBC is gallstone disease, the metaplasia-dysplasia-neoplasia sequence mostly explains gallbladder cancer development [5], and also explains that reactive atypia and dysplasia are more commonly encountered in histologic sections, when compared to GBC.

Some immunohistochemical (IHC) stains have been studied to aid the diagnosis of GBC. The p53 tumor suppressor gene is known to be involved in the carcinogenesis and prognostication of many human cancers, such as in the stomach, colon, and endometrium [6]. In its natural form, the wild-type p53 protein inhibits cell proliferation, whereas the mutated form is associated with oncogenic properties and accumulates in the nucleus. Some studies state p53 can be helpful in supporting a diagnosis of gallbladder carcinoma, though the reported rates of aberrant p53 staining range from 40–92% [6]. The insulin-like growth factor II mRNA binding protein 3 (IMP3) is a fetal oncoprotein involved in tumor cell proliferation, adhesion, and invasion. It is necessary for cellular migration and proliferation in fetal tissues, but normal adult tissues do not contain IHC-detectable levels of IMP3, making it a unique marker for oncologic study [7]. It has been suggested to be a marker for high-grade dysplasia in the biliary tract and have prognostic implications in biliary carcinomas [8]. S100P is a calcium-dependent signal transducer in the S100 protein family, which has been described in numerous carcinomas. Some have found a stepwise expression from reactive epithelium to low and high grade biliary intraepithelial neoplasia [9].

While these IHC stains have been studied in the context of biliary tract carcinomas, data is limited in evaluating reactive atypia versus dysplasia in gallbladder specimens. To the best of our knowledge, this is the first study to evaluate IHC stains with the aim to facilitate diagnosis of gallbladder reactive atypia and dysplasia. This study’s aim is to analyze the role of IMP3, p53, and S100P IHC stains as an adjunct to aid in differentiation of reactive atypia versus dysplasia in cholecystectomy specimens.

## Methods

### Case selection

This study was approved by the University of Texas Southwestern Medical Center Institutional Review Board. Fifty-four cholecystectomy specimens with a rendered final diagnosis of atypia (including reactive atypia) or neoplasia (including dysplasia and adenocarcinoma) between August 2019 and May 2021 were identified to be included in this study. Hematoxylin and eosin (H&E)-stained slides were screened and representative blocks from each case were selected by one gastrointestinal (GI) specialized pathologist. All slides were subsequently de-identified, randomized, and independently reviewed by 3 GI pathologists. Inclusion into the study required a consensus of the final diagnosis by all 3 pathologists. For any discrepant diagnosis, all 3 pathologists had a consensus meeting with agreement on the final diagnosis.

The diagnosis of dysplasia was established by the 5th edition of WHO for biliary intraepithelial neoplasia (BilIN). The cases were separated into five categories: normal epithelium (NL), reactive atypia (RA), low-grade dysplasia (LGD), high-grade dysplasia (HGD), and invasive adenocarcinoma (IAC). A total of 54 cases were selected for IHC staining, comprising 2 NL, 29 RA, 16 LGD, 2 HGD, and 5 IAC cases. All cases in the RA category were confirmed to have no evidence of local or distant malignant disease at 1–3 years of follow-up to support the designation of benignity. Patient demographic and clinical data were collected on all cases included in this study.

## IHC procedures

IHC detection of IMP3 was performed on sections of formalin-fixed, paraffin-embedded tissue from the selected cases. Antigen retrieval was performed at 97 °C, pH of 9.0 for 40 min. IMP3 antibody (M3626, clone 69.1, 1:100; Agilent, CA, USA) binding was performed at room temperature for 30 min. Final detection was performed using DAKO Envision Flex + HRP system (Agilent, CA, USA). Normal and neoplastic pancreas (pancreatic intraepithelial neoplasia/adenocarcinoma) were used as control tissue.

IHC detection of p53 was performed on sections of formalin-fixed, paraffin-embedded tissue from the selected cases. Analysis was performed on a Dako Autostainer Link 48 system. Briefly, the slides were baked for 20 min at 60 °C, then deparaffinized and hydrated before the antigen retrieval step. Heat-induced antigen retrieval was performed at 9.0 pH for 20 min in a Dako PT Link. The tissue was incubated with a peroxidase block and then an antibody incubation (Monoclonal Mouse Anti-Human p53 Protein Clone DO-7 Ready-to-use; Agilent, CA, USA) for 20 min. The staining was visualized using the EnVision FLEX visualization system. Normal colon and colonic adenocarcinoma were used as positive control, and normal stomach as negative control.

IHC detection of S100P was performed on sections of formalin-fixed, paraffin-embedded tissue from the selected cases. Immunohistochemical analysis was performed on a Dako Autostainer Link 48 system. Briefly, the slides were baked for 20 min at 60 °C, then deparaffinized and hydrated before the antigen retrieval step. Heat-induced antigen retrieval was performed at 6.0 pH for 20 min in a Dako PT Link. The tissue was incubated with a peroxidase block and then an antibody incubation (Recombinant Anti-S100P antibody 1:500 dilution; Abcam, MA, USA) for 20 min. The staining was visualized using the EnVision FLEX visualization system. Normal stomach was used as positive control, and normal pancreas as negative control.

### Evaluation of IHC staining

The IMP3, p53, and S100P IHC stains were performed and evaluated. For IMP3 and S100P stains, negative staining was defined as no immunoreactivity, or weak immunopositivity of any percentage, or moderate or strong immunolabeling of ≤ 10% of lesional cells. Positive staining was determined by moderate and/or strong immunolabeling of > 10% of lesional cells [8]. Positive staining for IMP3 was seen as cytoplasmic staining of cells, while S100P required either nuclear staining or nuclear and cytoplasmic staining. P53 was divided into wild-type expression or aberrant/mutant expression. Aberrant p53 was demonstrated as complete loss of nuclear staining (null pattern of staining) or increased nuclear staining, regarded as strong p53 staining compared to background level (overexpression), in keeping with the criteria used in Barrett’s mucosa [10]. IHC analysis was performed by two of the practicing GI pathologists with consensus achieved.

### Statistical analysis

Cases were additionally evaluated for positive staining for both IMP3 and p53 (“double stain”), and all three stains (“triple stain”). Chi-square test was used for statistical analysis. A p-value < 0.05 was considered statistically significant.

## Results

The 54 patients included in this study were mostly middle-aged women (average age: 44 years, age range: 19–87 years, 81% women and 19% male). The ethnicity of majority of patients was White Hispanic (80%, 43 of 54 cases), the remaining 20% being non-Hispanic (15% White; 5% Black). The average age was 44 years with a range of 19 to 87. Clinical indications for cholecystectomy included acute and chronic cholecystitis, cholelithiasis, and gallbladder polyp/mass.

**Fig. 1:**
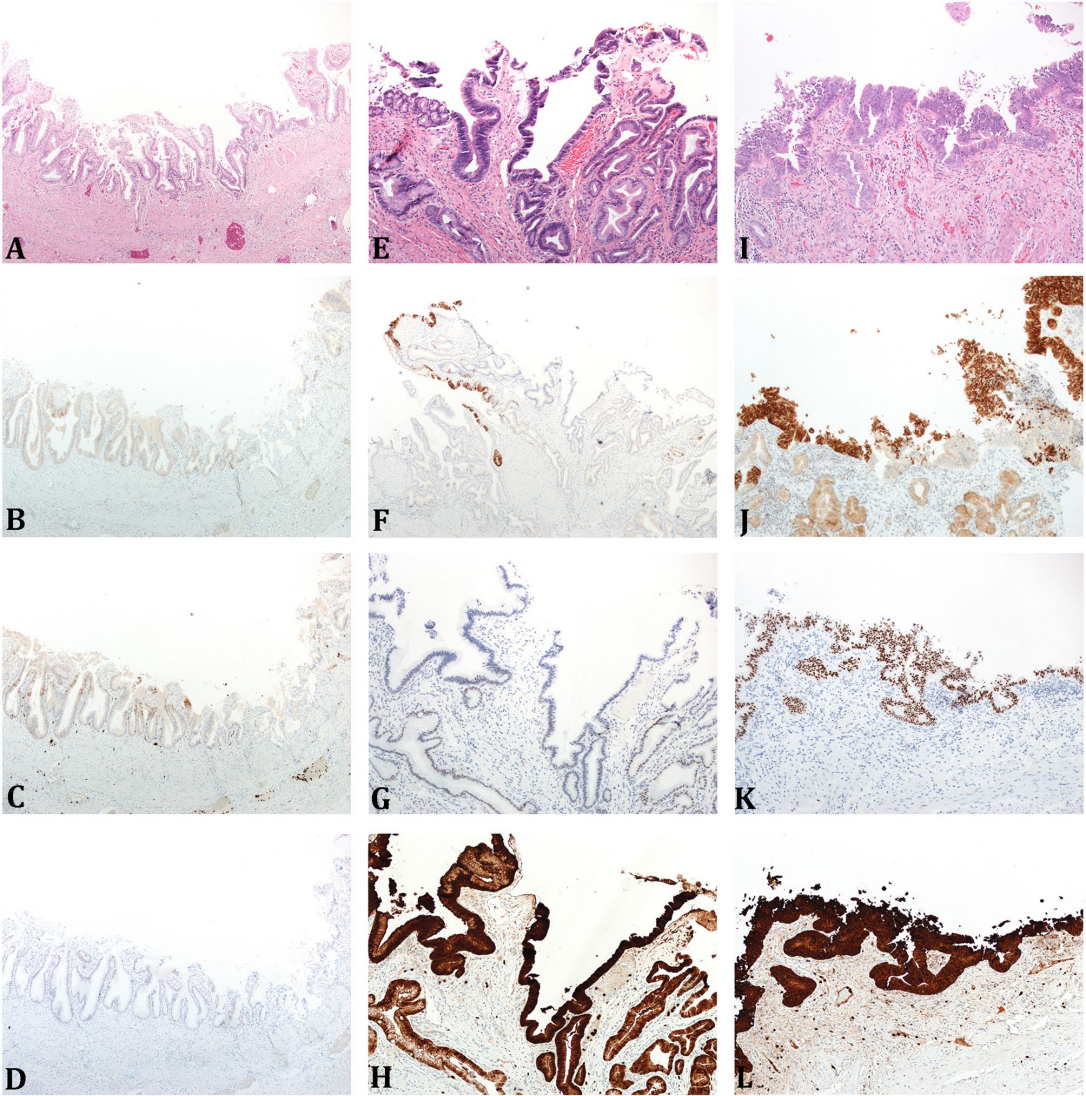
H&E, IMP3, p53, and S100P stains from representative gallbladder sections. **Legend**: H&E (**A**, **E**, **I**), IMP3 (**B**, **F**, **J**), p53 (**C**, **G**, **K**), and S100P (**D**, **H**, **L**) stains. Reactive atypia (**A**-**D**, 4X) shows weak IMP3 and S100P staining and wild-type p53. Low-grade dysplasia (**E**-**H**, 10X) shows IMP3 and S100P positivity, and null-staining aberrant p53. High-grade dysplasia (**I**-**L**, 10X) shows IMP3 and S100P positivity, with increased p53 aberrancy

The IHC staining pattern results for IMP3, p53, and S100P immunostains are tabulated in Table 1. All NL cases were negative for IMP3 and showed wild-type staining pattern with p53 immunostain. The majority of RA cases also showed IMP3 negativity (76%) and wild-type p53 (90%) staining pattern (Fig. 1). Specimens with dysplasia demonstrated higher rates of positive IMP3 staining and an aberrant p53 expression pattern. At least half of cases with LGD showed IMP3 positivity (56%) and aberrant p53 staining (50%) patterns (Fig. 1). Both cases with HGD showed IMP3 positivity (100%) and one of them also showed aberrant p53 staining (50%) pattern (Fig. 1). The majority of IAC cases also showed IMP3 positivity (80%) with aberrant p53 staining (100%). The differences in staining patterns of IMP3 and p53 stains were statistically significant between RA vs. LGD (*p* = 0.03 and 0.003, respectively), RA vs. LGD plus HGD (*p* = 0.01 and 0.002, respectively), but no significant differences were seen when comparing staining patterns between LGD vs. HGD cases, although we acknowledge that the numbers of HGD cases were low for a definitive interpretation (Table 1). S100P was expressed in most of the cases and showed no statistical significance among the diagnostic categories (Table 1).

**Table 1:** Immunohistochemical staining results for IMP3, p53, and S100P in selected gallbladder lesions

	IMP3		p53		S100P		IMP3/P53		IMP3/P53/S100	
	Negative/Weak	Positive	Wild-type	Aberrant	Negative/Weak	Positive	Non-double positive	Double positive	Non-triple positive	Triple positive
**NL n(%)**	2 (100%)	0 (0%)	2 (100%)	0 (0%)	1 (50%)	1 (50%)	2 (100%)	0 (0%)	2 (100%)	0 (0%)
**RA n(%)**	22 (75.9%)	7 (24.1%)	26 (89.7%)	3 (10.3%)	2 (6.9%)	27 (93.1%)	27 (93.1%)	2 (6.9%)	27 (93.1%)	2 (6.9%)
**LGD n(%)**	7 (43.7%)	9 (56.3%)	8 (50%)	8 (50%)	0 (0%)	16 (100%)	12 (75%)	4 (25%)	12 (75%)	4 (25%)
**HGD n(%)**	0 (0%)	2 (100%)	1 (50%)	1 (50%)	0 (0%)	2 (100%)	1 (50%)	1 (50%)	1(50%)	1(50%)
**IAC n(%)**	1 (20%)	4 (80%)	0 (0%)	5 (100%)	0 (0%)	5 (100%)	1 (20%)	4 (80%)	1 (20%)	4 (80%)
**p-value RA vs. LGD**	0.03		0.003		0.3		0.09		0.09	
**p-value RA vs. LGD + HGD**	0.01		0.002		0.3		0.05		0.05	

*Key* NL (normal epithelium); RA (reactive atypia); LGD (low-grade dysplasia); HGD (high-grade dysplasia); IAC (invasive adenocarcinoma)

Notably, when assessing IMP3 and p53 stains together, over 90% of RA cases (27 of 29 cases) showed IMP3 negativity with p53 wild-type pattern. When using IMP3 and p53 stains together (double stain), the specificity for neoplasia was 93.5%, despite its low sensitivity for detection (39.1%). However, no significant difference was found when using double stains (IMP and p53) for distinguishing between RA and LGD cases (*p* = 0.09) and between RA and LGD + HGD cases (*p* = 0.05). As the S100P stain demonstrated pan-positivity, the same statistical results were found for the triple stain as the double stain (Table 1).

## Discussion

We found that the use of p53 and IMP3 immunostains can be helpful in distinguishing reactive atypia from dysplasia, when dealing with these challenging case scenarios in cholecystectomy specimens. In particular, we found that negative IMP3 stain in conjunction with wild-type p53 staining would support a diagnosis of reactive atypia over true dysplasia.

GBC is considered to develop from a metaplasia-dysplasia-carcinoma sequence, making the diagnosis of dysplasia very important [5]. RA is a benign entity occurring in inflamed epithelium, but as dysplasia is frequently associated with chronic inflammation, dysplasia is often difficult to distinguish histologically from RA. Complete resection of gallbladder with dysplasia along with negative cystic duct resection margins is sufficient prophylactic treatment for GBC in these cases [11]. However, in addition to the possible progression of dysplasia to GBC, Rais et al. found that in cases with incidental gallbladder dysplasia, 18.9% of patients had an associated pancreatobiliary carcinoma [11]. Given the hypothesis of multifocal neoplastic potential in the pancreatobiliary tree, patients with dysplasia in the gallbladder would benefit from monitoring for development of other neoplasia of the biliary tract.

Wild-type p53 has functions in tumor suppression through cell cycle arrest, apoptosis, transcription, DNA repair, etc. It has a central role in preventing malignant progression, thus accumulation of genetic damage increases tendency for carcinogenesis [12]. It has previously been thought that p53 expression marked a late event in gallbladder carcinogenesis [13]. In a study by Legan et al., all NL and LGD gallbladder cases showed wild-type p53, whereas 31.2% of their HGD cases were p53 aberrant. They postulated that any p53 expression in LGD could be due to failure of p53 degradation, leading to accumulation of wild-type protein and thus pushing the cell into cycle arrest and apoptosis. These cells would not proliferate as malignant clones [14].

However, loss of a chromosomal region in the TP53 gene has been found in normal-appearing and dysplastic gallbladder epithelium, suggesting that TP53 abnormalities precede protein overexpression in the pathogenesis of GBC [12, 15]. P53 mutations have also been linked with dysplastic epithelium in patients with gallstone disease, indicating a chronic inflammatory cascade [16]. The results from a study by Wee et al. further reinforces this as they found 50% of dysplastic gallbladder tissue showed p53 aberrancy, with 28% expressing strongly (> 50%) [17]. In fact, a case report of a patient who had confirmed complete GBC resection post-cholecystectomy was found to have over-expression of mutant p53 in the normal surrounding epithelium of the cystic duct. This patient developed bile duct cancer only 2.5 years later [18]. Our study found 10% of RA, 50% of dysplasia, and 100% of IAC cases show mutated p53 on IHC. This supports the role that aberrant p53 has in gallbladder cancer pathogenesis as areas of strong staining could imply protein mutation has already occurred. In fact, it has been hypothesized that dysplasia with mutant p53 evolve into more aggressive tumor types with higher grade [19].

IMP3 expression in gallbladder carcinomas and dysplasia has shown contrasting findings. Kim et al. found negative IMP3 expression in dysplasia and chronic cholecystitis specimens while 87.7% of GBC expressed IMP3 immunoreactivity, concluding that IMP3 is a useful diagnostic marker for GBC. They also noted that strong IMP3 expression was associated with higher histological grade, advanced age, lymphatic invasion, and worse overall survival [20]. Some other studies have also supported similar findings of IMP3 predicting poor prognosis and the presence of invasion in biliary tract carcinomas [21, 22].

On the other hand, Riener et al. claimed IMP3 to be useful for HGD diagnosis in the extrahepatic biliary tract. In their study, all HGD demonstrated strong IMP3 staining, while all NL and LGD had weak or no IMP3 reactivity. This resulted in a sensitivity and specificity of 1 for IMP3 use in HGD in the extrahepatic biliary tract. They also found differences in IMP3 expressions in different types of cancers. GBC expressed IMP3 most strongly at 81.6%, whereas intra- and extra-hepatic cholangiocarcinoma showed IMP3 positivity rates of 36.8% and 50%, respectively [8]. While our study showed a similar IAC IMP3 expression of 80%, some RA cases in our study had positive IMP3 expression. We found that IMP3 is valuable in differentiating RA from LGD and HGD. These findings may be related to a difference in etiology and molecular alterations seen in gallbladder lesions compared to those from the rest of the biliary tract. There could be an earlier expression of IMP3 in the gallbladder compared to the extrahepatic bile ducts, or the IMP3 positive gallbladder cases, like those with mutant p53, are likely to become more aggressive later in their courses.

The utility of S100P in the gallbladder remains controversial. During embryonic development, S100P is expressed in several tissues, including the epithelium of the gallbladder [23]. In adults, S100P has been widely expressed in both normal and malignant tissues [24]. One study demonstrated negative S100P staining in NL gallbladder tissue and positive staining in 50% of chronic cholecystitis cases. They also noted positive staining of background stroma, inflammatory, and endothelial cells [25]. Another study found that S100P expression was significantly higher in GBC (61.7%) than in benign tissues [26]. However, the analysis from this present study indicates that S100P does not aid in gallbladder disease differentiation as the stain showed pan-positivity from NL to IAC tissue.

It is also worth mentioning that whether S100P should be used as a prognostic marker in GBC remains inconclusive. While one study found S100P to be associated with more advanced GBC disease and poor survival, their study was limited by the lack of a control group [27]. A meta-analysis conducted by Liu et al. concluded that S100P was not a helpful predictor of overall survival in patients with gallbladder cancer [28].

Due to the rare nature of gallbladder dysplasia and carcinomas, our study was limited by study size, especially the number of HGD specimens. A larger cohort with ample cases across all categories may provide stronger significance. Inevitably, interobserver variability in diagnosis among pathologists should also be noted, though we attempted to minimize this by requiring a consensus among the 3 GI subspecialized pathologists in this study. We also note that the cutoff ranges for a positive result vary from study to study, making direct comparisons difficult. A study in which the patients are followed longitudinally to assess for prognosis and development of neoplasia or worsening disease could provide greater insight into the role these proteins have on disease prediction and progression.

In summary, we conclude that IMP3 and p53 IHC stains showed statistically significant differences when differentiating RA from LGD and HGD, while S100P demonstrated no significant benefit. Morphology on H&E staining remains the standard for RA and dysplasia diagnosis; however, in cases that are difficult to distinguish, immunopositivity for IMP3 or p53 aberrant expression may help favor a diagnosis of LGD over RA.

## Data Availability

Data is provided within the manuscript or supplementary information files.

## Abbreviations

GBC: Gallbladder cancer
GI: Gastrointestinal
HGD: High-grade dysplasia
IAC: Invasive adenocarcinoma
IHC: Immunohistochemical
IMP3: Insulin-like growth factor II mRNA binding protein 3
LGD: Low-grade dysplasia
NL: Normal epithelium
RA: Reactive atypia
